# A Dual-Pathogen Mitral Valve Endocarditis Caused by *Coxiella burnetii* and *Streptococcus gordonii*—Which Came First?

**DOI:** 10.3390/pathogens12091130

**Published:** 2023-09-04

**Authors:** Ann-Sophie Kaemmerer, Francesco Ciotola, Walter Geißdörfer, Frank Harig, Jochen Mattner, Timo Seitz, Mathieu N. Suleiman, Michael Weyand, Christian Heim

**Affiliations:** 1 Department of Cardiac Surgery, University Hospital Erlangen, Friedrich-Alexander-University Erlangen-Nürnberg, D-91054 Erlangen, Germany; frank.harig@uk-erlangen.de (F.H.); timo.seitz@uk-erlangen.de (T.S.); mathieu.suleiman@uk-erlangen.de (M.N.S.); michael.weyand@uk-erlangen.de (M.W.); christian.heim@uk-erlangen.de (C.H.); 2 Department of Cardiology and Pneumonology (Med 1), Klinikum Fürth, Academic Teaching Hospital of the Friedrich-Alexander-University Erlangen-Nürnberg, D-90766 Fürth, Germany; francesco.ciotola91@gmail.com; 3 Institute of Microbiology—Clinical Microbiology, Immunology, Hygiene, University Hospital Erlangen, Friedrich-Alexander-University Erlangen-Nürnberg, D-91054 Erlangen, Germany; walter.geissdoerfer@uk-erlangen.de (W.G.); jochen.mattner@uk-erlangen.de (J.M.)

**Keywords:** polymicrobial infective endocarditis, dual-pathogen endocarditis, blood-culture-negative endocarditis, minimally invasive mitral valve replacement, *Coxiella burnetii*, *Streptococcus gordonii*, Q fever, endocarditis

## Abstract

Infective endocarditis (IE) is still a life-threatening disease with high morbidity and mortality. While usually caused by a single bacterium, poly-microbial infective endocarditis (IE) is rare. Here, we report a (blood-culture-negative) dual pathogen mitral valve IE caused by *Coxiella burnetii* and *Streptococcus gordonii*: A 53-year-old woman was presented to an internal medicine department with abdominal pain for further evaluation. Within the diagnostic work up, transthoracic echocardiography (TTE) revealed an irregularly shaped echogenic mass (5 × 13 mm) adherent to the edge of the posterior mitral valve leaflet and protruding into the left atrium. As infected endocarditis was suspected, blood cultures were initially obtained, but they remained negative. Chronic Q fever infection was diagnosed using serologic testing. After the occurrence of cerebral thromboembolic events, the patient was admitted for mitral valve surgery. Intraoperatively, a massively destructed mitral valve with adhering vegetations was noted. Examination of the mitral valve by broad-range bacterial polymerase chain reaction (PCR) and amplicon sequencing confirmed *Coxiella burnetii* infection and yielded *Streptococcus gordonii* as the second pathogen. Based on the detailed diagnosis, appropriate antibiotic therapy of both pathogens was initiated, and the patient could be discharged uneventfully on the 11th postoperative day after a successful minimal-invasive mitral valve replacement.

## 1. Introduction

Infective endocarditis (IE) is an inflammation of cardiovascular structures, caused by microorganisms, mainly bacteria. Even with appropriate diagnosis and therapy, it is still a life-threatening disease with high morbidity and mortality [1]. The incidence of IE is between 2 and 5 cases per 100,000 persons per year, with a slight increase in Western countries, mainly due to “healthcare-associated” or nosocomial endocarditis [2,3]. 

If left untreated, infective endocarditis is often lethal. However, despite a contemporary treatment, including antibiotics and early surgery, the mean in-hospital mortality of IE is 15–20% with a 1-year mortality of approximately 40% [4].

Predilection sites for IE are the heart valves, the mural endocardium, cardiac vessels, and implanted foreign material, such as artificial heart valves or pacemaker leads. 

Predisposing factors for IE are acquired heart valve disease, congenital heart defects, temporary or permanent intravenous catheters, cardiac pacemakers, implanted cardioverters, hemodialysis, ports, intravenous (i.v.) drug abuse, or a previous history of IE. However, in 30 to 50%, no predisposing risk factors can be detected [2,3,5].

The spectrum of the bacteria causing IE has changed in recent decades. *Streptococci* were responsible for the majority of IE until the end of the 20th century; *staphylococci* are nowadays the most frequent microbiological etiology of IE in Western high-income countries [6,7,8]. Currently, *staphylococci* and *streptococci* are responsible for 80–90% of IE cases, *enterococci* for 10%, and bacteria of the HACEK group (*Haemophilus species*, *Actinobacillus actinomycetemcomitans*, *Cardiobacterium hominis*, *Eikenella corrodens*, *Kingella kingae*) or fungi are less frequent. The proportion of culture-negative IE is up to 10% [9].

Blood culture-negative endocarditis (BCNE), *viridans group* endocarditis, and *Coxiella burnetii* endocarditis are three distinct types with unique characteristics. 

BCNE refers to cases where standard blood cultures fail to identify the causative microorganism, often necessitating alternative diagnostic methods. 

*Viridans group* endocarditis is caused by a collection of bacteria commonly found in the oral cavity, and its clinical course can vary from mild to severe. 

*Coxiella burnetii* endocarditis is caused by the bacteria *Coxiella burnetii*, which is a challenging-to-diagnose condition. 

Non-infectious etiologies (e.g., rheumatic fever, Libman–Sacks-Endocarditis in systemic lupus erythematodes, phospholipid antibody syndrome, rheumatoid arthritis, vasculitis, post-radiation endocarditis, etc.) should be considered in the differential diagnosis of infectious diseases [9,10].

The diagnosis of IE is nowadays based on clinical presentation; clinical suspicion; culture-based pathogen detection; echocardiography; and, if necessary, further diagnostic procedures. The modified Duke criteria can be used to establish the diagnosis of endocarditis based on clinical, microbiologic, and echocardiographic criteria [11].

Clinical diagnosis is extended by pathogen detection from blood cultures. If difficult-to-cultivate pathogens are suspected, anamnestic evidence of risk factors for rare pathogens must be considered, such as travel history, diet, animal contact, etc. For special cases, serological or antigen tests, as well as molecular biological methods, such as PCR amplification with subsequent sequencing, are available. In addition, biopsy material from peripheral emboli or tissue taken intraoperatively from the heart can be examined [9,12,13,14].

BCNE can be classified into three main categories: (a) bacterial endocarditis with blood cultures sterilized by previous antibacterial treatment, usually due to common endocarditis-causing bacteria, i.e., *streptococci* or *staphylococci*; (b) endocarditis related to microorganisms that require sophisticated diagnostic procedures, e.g., HACEK bacteria or nutritionally variant *streptococci*, such as *Granulicatella* or *Abiotrophia sp*.; (c) and “true” blood culture-negative endocarditis, caused by intra-cellular bacteria, such as *Bartonella*, *Coxiella* or *Tropheryma whipplei*, which cannot be routinely cultured with currently available techniques and require molecular biological detection methods [15,16,17,18]. 

BCNE accounts for up to 20% of infective endocarditis. While the most common cause of BCNE remains the initiation of antibiotics prior to culture, intracellular organisms, such as *Tropheryma whipplei* or *Coxiella* and *Bartonella* spp., account for a significant proportion of cases [19]. Indeed, endocarditis is the most common presentation of chronic Q fever, a zoonosis caused by the obligate intracellular bacterium, *Coxiella burnetii* [20]. Approximately two-thirds of patients with chronic Q-fever experience endocarditis [21]. 

Diagnosis of endocarditis caused by *Coxiella burnetti* requires 16-S ribosomal ribonucleic acid (rRNA) bacterial polymerase chain reaction (PCR) analysis of affected valve material and serological testing to confirm chronic Q fever. 

*Viridans streptococci* are part of the normal flora of the mouth. They can be responsible for dental caries and pericoronitis, as well as for subacute infective endocarditis due to bacteremia [22]. 

Diagnosis is usually confirmed by positive blood cultures, gram stains, and culture or molecular analysis of the affected heart valve. The clinical presumptive diagnosis is confirmed by echocardiography.

Nowadays, the causative microorganism is identified in approximately 90% of the episodes of IE [2,23]. The isolation of more than one microorganism in patients with IE is quite uncommon, ranging from 1% to 6.8% [24,25].

As IE is usually caused by a single bacterium, a dual pathogen IE, caused by *Coxiella burnetii* and *Streptococcus gordonii*, as presented here, is rare. Therefore, we report this case of complicated mitral valve endocarditis and emphasize the importance of clinical, microbial and echocardiographic diagnosis, laboratory tests, and appropriate medical and surgical treatment.

## 2. Case Presentation

A 53-year-old woman (62.2 kg, 173 cm, BMI: 20.8 kg/m^2^) from Eastern Europe, working as a professional cook, initially presented in a Romanian hospital with a complex history that spanned approximately 6 months. Due to severe headache, forgetfulness, and visual disturbances, a stroke was suspected, but not further investigated.

A residual gait disorder remained after the cerebral event. After discharge from the hospital, the patient developed unbearable abdominal pain and a high fever for about 2 weeks. Therefore, she was readmitted to a Romanian hospital and operated on for acute acalculous cholecystitis complicated with perforation accompanied by peritonitis. 

Two months after the cholecystectomy, the patient was presented to a regional German hospital with radiating flank pain for further evaluation. Abdominal sonography revealed no local abscess. A colonoscopy showed diverticulosis throughout the colon and mild diverticulitis. Taking into account the previous suspected diagnosis of a cerebrovascular event, a carotid Doppler was performed, which showed no relevant carotid stenosis. In addition, transthoracic echocardiography was performed, which revealed a floating structure at the posterior mitral valve leaflet with a resulting moderate-to-severe mitral valve regurgitation. The left ventricle was dilated, and the systolic function of the left ventricle was normal. 

**Figure 1:**
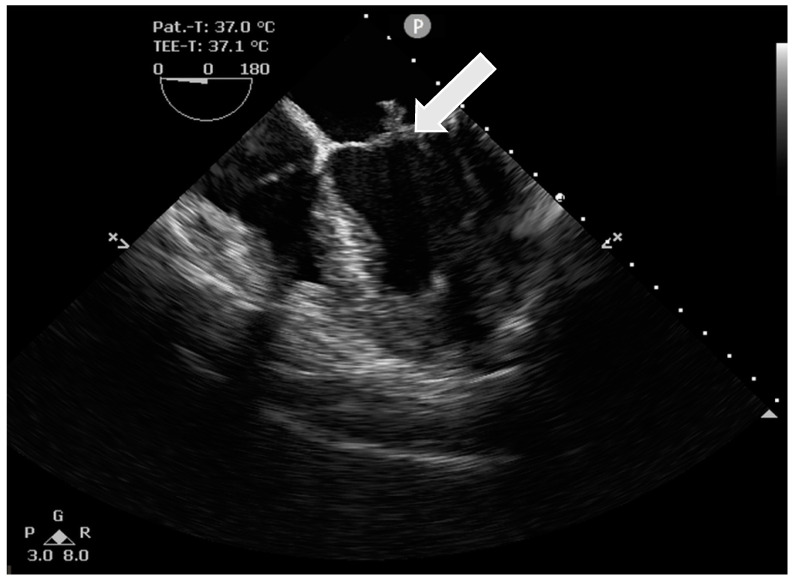
Intraoperative transesophageal echocardiography demonstrating the vegetations of the mitral valve. (arrow: vegetation).

**Figure 2:**
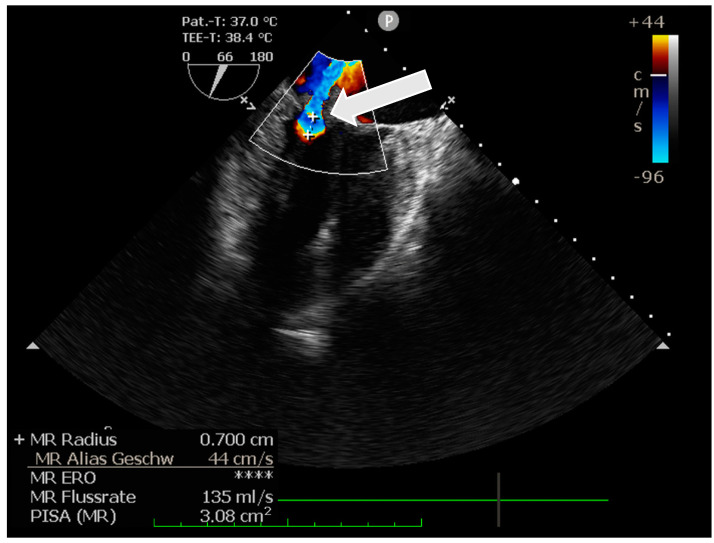
Intraoperative transesophageal echocardiography demonstrating the mitral valve insufficiency. (arrow: insufficiency).

The intraoperative transesophageal echocardiography confirmed the presence of a floating structure at the leading edge of the posterior mitral valve leaflet measuring 13 × 6 mm, consistent with an endocarditic vegetation (Figure 1), as well as the previously described mitral insufficiency (Figure 2). No pre-existing heart (valve) disease of the patient and no history of immunosuppression were known. 

**Figure 3:**
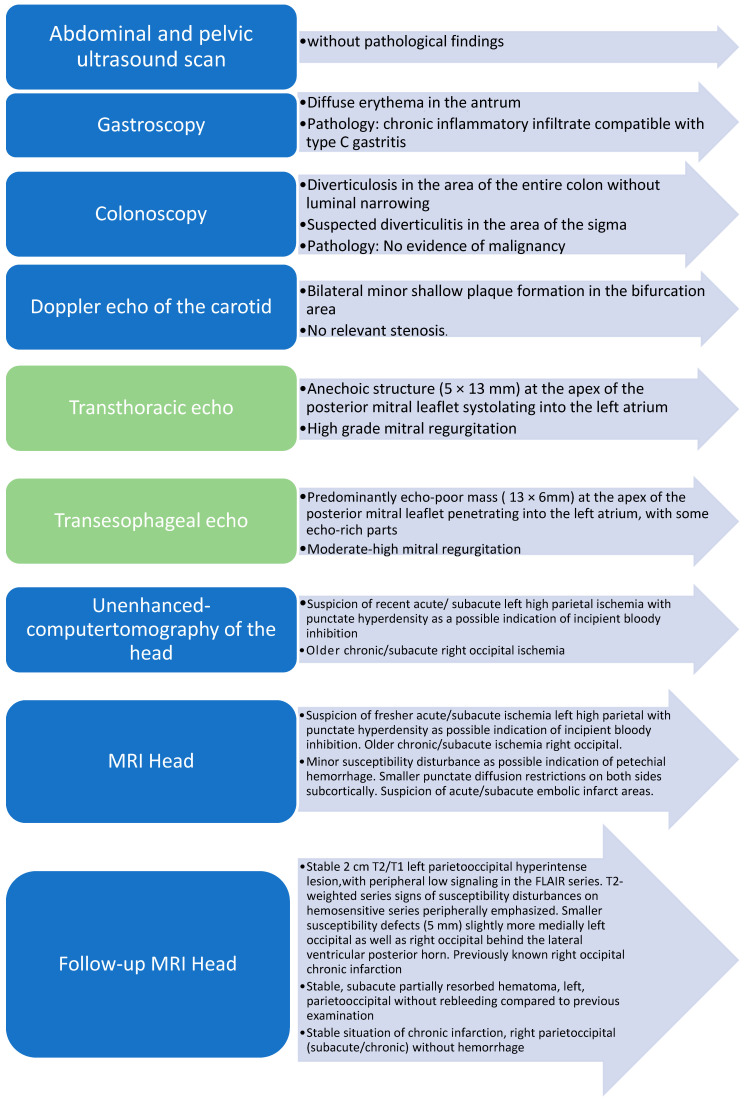
Patient’s diagnostics during hospitalization; green indicating: signs of infective endocarditis. (echo:_echocardiography, MRI: magnetic resonance imaging).

The patient was always afebrile, and the inflammatory markers were only slightly above the normal range. After 8 days of hospitalization with no improvement in the patient’s condition, two pairs of blood cultures were obtained prior to initiation of anti-infective treatment and found to be sterile. Extended serological testing for atypical endocarditis yielded significant positive titers for chronic *Coxiella burnetii* infection (Table 1), (Figure 3). Serological testing for *Bartonella henselae*, *Bartonella quintana*, *Legionella pneumophilia*, as well as *Mycoplasma pneumoniae* were additionally obtained. *Bartonella* spp. was excluded as a potential cause of BCNE. Although rarely reported with 3 to 4 reports ion the literature, *Mycoplasma pneumoniae* can cause BCNE as well. However, *M. pneumoniae* was excluded as was *Legionella pneumophila*, a well-known but rare cause of bacterial endocarditis.

Accordingly, a targeted anti-infective treatment with doxycycline and hydroxychloroquine was initiated. 

Because of the worsening mitral regurgitation, the floating structure at the mitral valve leaflet and the assumption that the previous neurologic symptoms were related to septic cerebral embolism, the patient was referred to our heart surgery clinic for further surgical treatment. The surgery was urgently planned with a class I B recommendation according to the current ESC Guidelines due to the size and embolic risk of the IE and due to the severe mitral valve regurgitation [26]. 

There were no symptoms suggestive of systemic infection, such as anorexia, headache, and generalized weakness, and no symptoms that would raise concern for aortic or mitral valve insufficiency, such as chest pain, dyspnea, decreased exercise tolerance, orthopnea, or paroxysmal nocturnal dyspnea. 

The clinical examination showed no classic immunologic and hemorrhagic cutaneous sequela of IE, no Osler nodes (painful subcutaneous nodules typically found on the palm), no subungual splinter hemorrhages, and no Janeway lesions (painless hemorrhagic plaques on the palms/soles). The abdominal exam revealed no splenomegaly or even localized peritonitis, and no signs of intracerebral embolization were present.

The patient`s preoperative medical history revealed a history of a cerebrovascular event with residual gait disorder, an acalculous cholecystitis complicated by perforation with peritonitis, and mild diverticulitis.

No cardiovascular risk factors were present, and the patient did not suffer from diabetes mellitus, dyslipidemia, and did not have a history of hypertension. 

The patient was a smoker but denied using alcohol or illicit drugs. She lived with 3 cats and a dog in the Romanian countryside, and that free-roaming sheep occasionally stayed near her property. 

The electrocardiography showed a sinus rhythm, 64 bpm, axis: vertical heart position, P-waves: present, normal physiology, PR Interval: normal, no pathological Q waves, QRS complexes narrow, and no ST segment changes.

Her blood pressure was 130/86 mmHg normal, her body temperature was 36.6 °C.

On the day of admission, the following blood values were abnormal: Gamma-GT 53 U/L (reference: <40 U/L), ASAT 37 U/L (reference: <35 U/L), ALAT 38 U/L (reference: <35U/L), alkaline phosphatase 163 U/L (reference:35–105 U/L), and C-reactive protein (CRP) 11.6 mg/L (reference: <5 mg/L).

Preoperatively, computed tomography of the aorta and the inguinal vessels was performed to ensure access routes for peripheral extracorporeal circulation and to exclude anatomic contraindications for a minimally invasive mitral valve replacement. 

For this procedure, the femoral artery and vein were percutaneously cannulated using the Seldinger technique for extra corporeal circulation. A 4 cm right mini-thoracotomy incision was performed at the level of the 5th intercostal space for the insertion of the flexible tissue retractor and a trocar was introduced in the 3rd intercostal space for the 0° 3D endoscope [27]. 

The minimally invasive surgical technique was chosen because it is less invasive, causes less trauma to the chest and surrounding tissues, and usually results in a faster postoperative recovery.

**Figure 4:**
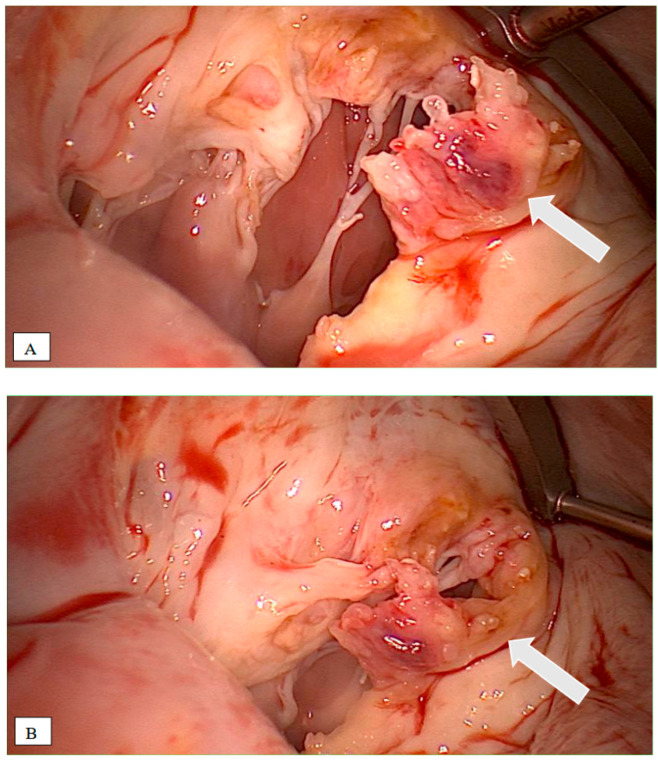
(**A**,**B**): Intraoperative picture of the mitral valve; arrow: indicating vegetations.

Intraoperatively, a completely destroyed mitral valve was seen, with the IE extending into the anterior mitral leaflet and the subvalvular holding apparatus (Figure 4A,B). 

The infected parts of the mitral valve were completely resected and sent for microbiological examination. The lateral parts of the subvalvular apparatus could be preserved and a mechanical 31 mm mitral valve prosthesis (St. Jude Mechanical heart valve SJM Masters Series 31 mm) was implanted. After atrial closure and de-aring, intraoperative transesophageal echocardiography showed a well-functioning mitral valve prothesis. 

Postoperatively, the patient was transferred hemodynamically stable under low doses of catecholamine to the intensive care unit and could be transferred to the normal ward on POD 1.

Histopathological examination revealed a mitral valve with preserved trilaminar structure, fibrin exudation, and extensive necrosis. There was predominantly lymphoplasmocytic; focally, also mild florid histiocytic granulomatous inflammation, including giant cells. These findings are compatible with florid IE [28]. 

Microbiological examination showed a moderate accumulation of leukocytes in the Gram stain of the removed heart valve. No bacteria or other microorganisms were detectable. Bacterial cultures of the resected material remained negative for 6 days of incubation, without bacterial growth.

**Figure 5:**
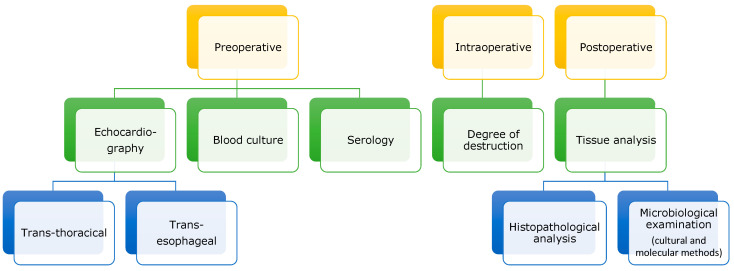
Diagnostic steps of infective endocarditis in a surgical setting.

Broad-range PCR for bacterial DNA (16S rRNA gene amplification) from the explanted valve was positive, and sequence analysis using a combination of universal, Gram-negative- and Gram-positive-specific sequencing primers revealed a mixture of *Coxiella burnetii* and *Streptococcus gordonii* (Figure 5).

*Streptococcus gordonii* as a member of the *viridans streptococci* is a common inhabitant of the oral flora. Since the dental status of the patient required treatment, we considered the detection in the valve material, of *Streptocococcus gordonii*, a common cause of endocarditis, a true finding, and excluded it as a contaminant. 

Based on these findings, additional intravenous ceftriaxon therapy for *Streptococcus gordonii* was initiated immediately. This was due to the unclear involvement of neurologic symptoms suggestive of a neurological event in the past, which was probably a septic embolic stroke. Additionally, a cholecystectomy and a not completely rehabilitated tooth status, as well as a mitral valve replacement, and a 4-week course of intravenous ceftriaxone was chosen as a precaution to avoid biofilm formation.

The dental examination revealed a dental condition requiring treatment. Furthermore, teeth as a potential port of entry for bacteria of the oral commensal flora was suspected, but upon further examination, this was considered unlikely. Thus, only further monitoring without additional surgical procedures was recommended. 

After a complication-free course, the patient was discharged on the 11th postoperative day. However, the patient had to remain under close surveillance. This includes the targeted anti-infective treatment with an 18-month oral anti-infective therapy consisting of a combination of doxycycline and hydroxychloroquine due to the detection of *Coxiella burnetii*, as well as a 4-week intravenous therapy with ceftriaxon due to the concomitant detection of *Streptococcus gordonii* on the valve. 

The postoperative transthoracic echocardiography showed a preserved left ventricular pump function (Ejection fraction (EF) of 61% using Simpson’s biplane method for the calculation of EF) with a mild left ventricular dilatation (Left Ventricular End Diastolic Diameter (LVEDD) (53 mm without LV hypertrophy (Interventricular Septal Thickness at Diastole (IVSD) (0.9 cm). The left atrium was normal in size (volume/body surface area (BSA): 23 mL/m^2^), thus, declining in volume compared with preoperative findings. The right ventricle was normal in size (basal diameter 3.4 cm) and well contractile (Tricuspid Annular Plane Systolic Excursion (TAPSE) (1.9 cm). The right atrium was normal in size. There was no evidence of pulmonary hypertension. No significant valvular dysfunction of the aortic; tricuspid and pulmonary valve could be detected. The artificial valve in mitral position (Mechanical Master 501 Abbott 31 mm) showed a good leaflet opening and no evidence of stenosis or Prosthesis-patient mismatch (PPM) (mean gradient 2 mmHg, effective orifice area (EOA) by continuity equation 2.44 cm^2^) with a mild transvalvular regurgitation. A minimal circular pericardial effusion (5 mm); hemodynamically irrelevant was seen. The aortic root measured 2.7 cm.

The diagnosis of infectious endocarditis due to *Coxiella burnetii* in the presented case was based on the echocardiographic findings (transesophageal), which was performed as part of the stroke evaluation. Because blood cultures remained negative, a serologic test for chronic Q fever was performed in the context of suspicious medical history and confirmed using PCR from heart valve material.

### 2.1. Follow-Up Recommendations

A serological control every 3 months and an ophthalmological control examination due to the intake of hydroxychloroquine, as well as level controls of hydroxychloroquine every 3 months (target 0.8–1.2 mg/L) were recommended by the institutional physicians. Furthermore, close monitoring of teeth and if necessary, tooth extraction was recommended by the institutional dentists. 

### 2.2. Postoperative Event

The patient was readmitted to the hospital 3 months later after three consecutive tonic-clonic seizures, which the neurologists classified as status epilepticus. The cranial-CT examination performed revealed an old posterior partial infarction and a medial partial infarction. EEG examination revealed left temporal dysfunction. A structural epilepsy was assumed. Anticonvulsant therapy was started with levetiracetam due to the history of cardiac disease. The patient was well enough to be discharged and a follow-up with a neurologist and regular EEG studies were arranged.

## 3. Discussion

To our best knowledge, this is the first case reporting a culture-negative infective endocarditis (IE) with *Coxiella burnetii* and *Streptococcus gordonii*. 

Most cases of IE have a bacterial infectious etiology. Currently, *staphylococci* and *streptococci* are responsible for 80–90% of IE cases, *enterococci* for 10%, and bacteria of the HACEK group (*Haemophilus*, *Aggregatibacter*, *Cardiobacterium*, *Eikenella*, *Kingella*) and fungi are less frequent. The proportion of culture-negative IE is up to 10% [3,9].

Usually, in almost 90% of the cases, IE is caused by a single bacterium [2]. In contrast, poly-microbial IE, as described in the current case report, is rare, accounting for 1 to 6.8% of cases [29]. 

Polymicrobial endocarditis relating to Q fever is even rarer [30,31]. All described cases had coinfection with a typical endocarditis-causing pathogen: *Streptococcus*, *Enterococcus*, and *Staphylococcus* spp. [31,32,33] In particular, the combination of *Coxiella burnetii* and *Streptococcus gordonii* as causative agents of IE has not been reported before despite *Streptococcus gordonii* being commonly found in the oral cavity. 

*Coxiella burnetii*, the pathogen of Q fever, causes IE in up to 5% in some countries. According to the European Centre for Disease Prevention and Control, only 109 confirmed Q fever cases were registered in 2019 in Romania [34].

Q fever is a worldwide zoonosis and was first described in 1935 after an outbreak in slaughterhouse workers in Queensland, Australia. Reservoirs of the bacteria are symptomless domestic and wild animals, in particular sheep. Infection usually occurs by inhalation of infectious dust-containing bacterial cells showing long-term viability in the environment. The formation of highly infective spore-like cells led to the classification of *C. burnetii* as a level 3 biosafety microorganism [35,36]. 

Infection via ticks and food is also possible. The affected are particularly men over the age of 40 years, immunocompromised patients, or pregnant women. 

The incubation period for Q fever is about 14–21 days. Symptoms range from asymptomatic to severe courses with high fever, eye infections, respiratory infections, and severe headaches. In some cases, like in our patient, Q fever can be associated with acute acalculous cholecystitis [37]. Chronic courses are characterized by inflammation of the heart valves, hepatitis, or involvement of other organs. The risk of embolic events in the setting of Q fever is described as 33% (1/3). Mortality rates are up to 20%. 

Endocarditis due to *C. burnetii* manifests as a culture-negative endocarditis because *Coxiella burnetii* is an obligate intracellular pathogen, which cannot be detected by routine blood culture, leading to delayed diagnosis [38,39,40,41]. 

*Coxiella burnetii* does not grow in blood cultures nor on agar plate media, in fact, it is a level 3 biosafety microorganism and very few research laboratories are able to grow *C. burnetii* in cell cultures worldwide [35,36].

Additionally, serologic testing should be performed when routine blood culture testing does not yield an etiologic diagnosis. Systemic pathologic and microbial examination of excised heart valves, including broad-spectrum PCR, should be performed [38,42,43,44].

In the present case, the diagnosis of Q fever was first established serologically. However, the patient’s medical history could have been indicative. The patient is a meat processing professional cook, living with three cats and a dog in the countryside in Romania. She reported that free-roaming sheep occasionally stayed close to her property. The reported weight loss of about 20 kg within 4 months is striking but not diagnostic. This also applies to the other symptoms that occurred, such as headache and visual disturbances, which are not pathognomonic.

Cases of simultaneous or consecutive infections with *C. burnetii* and agents of other tickborne diseases, such as *Rickettsia conorii* (2 patients), *Rickettsia slovaca* [2], *Rickettsia africae* [1], and *Francisella tularensis* [1] have been described [45].

There is one search of the literature for articles on coinfection involving *C. burnetii*, but also *Brucella* spp. and *Rift Valley fever virus* (RVFV) in humans and terrestrial animals from 2004 to August 2020. A total of 50 pathogens were reported to coinfect with *C. burnetii*, *Brucella* spp., or RVFV. Most of the coinfecting pathogens were bacteria, but parasites and viruses were also identified as coinfecting [46].

Indications for cardiovascular implantable electronic devices (CIEDs) such as permanent pacemakers (PPMs) and implantable cardioverter defibrillators (ICDs) have increased in recent decades. While *staphylococci* are the main cause of CIED infections, there are only isolated reports of CIED infections by the Q-fever agent [47]. 

*Streptococcus gordonii* is a Gram-positive, alpha-hemolytic species from the *Viridans streptococci* and a primary colonizer of the tooth surface, usually considered commensal, which is responsible for plaque formation and associated with dental caries [48]. Once in the bloodstream, *Streptococcus gordonii* appears to express/acquire virulence factors that are pathogenic in the development of IE [49]. *Streptococcus gordonii* can cause infective endocarditis, particularly after manipulations at the oral mucosa even in immunocompetent individuals, particularly after tooth extractions or oral surgery, but also after everyday situations, such as brushing teeth, chewing, or flossing. Patients with deficient immune responses are particularly endangered [50].

IE caused by *Streptococcus gordonii* occurs in app. 1.7–6.2 per 100,000 patients annually in the USA and Europe and has an estimated mortality rate of 40% [51].

In the present case, *Streptococcus gordonii* was missed in the blood cultures and the intraoperatively obtained valve material exclusively was detected using broad-range PCR and amplicon sequencing (microbiological smears and culture negative). The fact that the blood cultures were negative may have been due to the previous antibiotic treatments [52,53,54]. Although the present hospitalization blood cultures have been obtained prior to the doxycycline treatment for Q fever, the patient was probably treated with antibiotics during the preceding 6-month hospital history in Romania.

Since pre-existing heart disease was denied by the patient and no clinical signs for it were found during diagnostic procedures, infection with one of the pathogens promoted the pre-disposition for the infection with the second one. Which one of the bacteria was first, however, could only be speculated. Although endocarditis during acute infection with *Coxiella burnetii* is an emerging clinical entity observed in adults that has been attributed to an autoimmune complication of early infection [55,56,57], *Coxiella* endocarditis develops usually only after months of infection and does usually not cause vegetations on heart valves. Thus, vegetations might be rather a consequence of *Streptococcus gordonii* infection. However, these vegetations might have underlied the suspected thromboembolic events in the past as well, suggesting that dual infection with both bacteria might have lasted for several months [10]. 

### Minimally Invasive Mitral Valve Replacement in Infective Endocarditis

The beginning of minimally invasive cardiac surgery dates back to the early 1990s, with the first steps being taken by mitral valve replacement [58,59]. 

The indication for surgical intervention in IE is still a matter of debate [1]. However, according to the literature, blood culture-positive IE operative treatment is superior to IE in which the pathogen has not been identified by blood cultures. Of particular importance is the fact that during surgery, tissue material can be obtained for further microbiological and pathological processing; thus, obtaining evidence of the pathogen and starting a targeted antibiotic therapy. 

A recent systematic review of minimally invasive mitral valve replacement in IE showed that in experienced hands, the minimally invasive approach can be successful, has acceptable perioperative morbidity as well as short- and intermediate-term mortality, and should be considered an acceptable alternative to sternotomy in IE of the mitral valve [60].

## 4. Conclusions

IE has still a high morbidity and mortality. Therefore, correct and early diagnosis and treatment are crucial for the prognosis. 

Usually, IE is caused by a single bacterium, but the proportion of culture-negative IE accounts for up to 10%. Poly-microbial IE, as described in the current case report, is quite rare. In particular, the combination of *Coxiella burnetii* and *Streptococcus gordonii* as causative agents of IE is exceptional. 

The presented case proves that atypical pathogens should always be considered in suspected IE. Particularly with negative blood cultures, further extended diagnostic procedures and tests should be employed to avoid a fatal course of IE and in order to start targeted therapy. 

The presented case underlines the option and advantage of minimally invasive surgery in IE of the mitral valve. 

As the prognosis of IE deeply depends on early diagnosis, interdisciplinary cooperation between representatives of cardiology, cardiac surgery, infective disease, microbiology, and pathology departments is of utmost importance. Affected patients with IE are cared for in a tertiary care center with a dedicated expert team [61]. It should also be noted that an infection with multiple agents is possible. In the case of positive blood cultures, in everyday clinical practice, often no further diagnostic tests are performed. However, in special cases where patients do not experience any clinical improvement despite targeted therapy when germs are detected, diagnostic tests should be repeated to check for further evidence of further pathogens and an infection with multiple germs should at least be ruled out.

Lastly, we would like to emphasize that regular follow-ups of such patients are of utmost importance.

## Figures and Tables

**Table 1:** Patient’s serology examination results.

	Results	Unit	Evaluation	Reference Value
**Enzyme immunoassay (EIA) method**				
Phase I-Ag IgG	2.2	Ratio	Elevated	<0.8
Phase II-Ag IgG	1.5	Ratio	Elevated	<0.8
Phase II-Ag IgM	1.0	Index	Borderline	<0.9
**Immunofluorescence test (IIFT) method**				
Phase I-Ag IgG	1:40,960	Titer	Highly elevated	<1:20
Phase I-Ag IgM	1:40,960	Titer	Highly elevated	<1:10
Phase II-Ag IgG	1:10,240	Titer	Highly elevated	<1:80
Phase II-Ag IgM	1:320	Titer	Highly elevated	<1:10
**The serological findings indicate a chronic** ***Coxiella burnetii*** **infection.**				

## Data Availability

Data sharing is not applicable to this article as no datasets were generated or analyzed during the current study.
